# Recurrent genetic alterations in epigenetically defined pineoblastoma subtypes

**DOI:** 10.1186/s40478-025-02140-7

**Published:** 2025-11-25

**Authors:** Tobias Goschzik, Mathias Yuan, Elke Pfaff, Manuel E. B. Müller, Martin Mynarek, Evelyn Dörner, David T. W. Jones, Stefan M. Pfister, Stefan Rutkowski, Torsten Pietsch

**Affiliations:** 1 Department of Neuropathology, University of Bonn Medical Center, Venusberg-Campus 1, 53127 Bonn, Germany; 2 Department of Pediatric Hematology/Oncology, University Clinics Hamburg-Eppendorf, Hamburg, Germany; 3 Division of Pediatric Glioma Research, Hopp Children’s Cancer Center Heidelberg (KiTZ), Heidelberg, Germany; 4 National Center for Tumor Diseases (NCT), NCT Heidelberg, a Partnership Between DKFZ and Heidelberg University Hospital, Heidelberg, Germany; 5 German Cancer Research Center (DKFZ), Heidelberg, Germany; 6 Department of Pediatric Oncology, Hematology, Immunology and Pulmonology, Heidelberg University Hospital, Heidelberg, Germany; 7 Mildred Scheel Cancer Career Center HaTriCS4, University Medical Center Hamburg-Eppendorf, Hamburg, Germany; 8 Hopp Children’s Cancer Center Heidelberg (KiTZ), Heidelberg, Germany; 9 Division of Pediatric Glioma Research (B360), German Cancer Research Center (DKFZ), Heidelberg, Germany; 10 Division of Pediatric Neurooncology, German Cancer Research Center (DKFZ), German Cancer Consortium (DKTK), Heidelberg, Germany; 11 DGNN Brain Tumor Reference Center, University of Bonn Medical Center, Bonn, Germany

**Keywords:** Pineoblastoma, Epigenetic subtypes, *DICER1*, *DROSHA*, PPTID

## Abstract

Previous studies have revealed four distinct epigenetic consensus pineoblastoma (PB) subtypes. The aim of this study was to confirm and further extend their respective genetic underpinnings. Cytogenetics of 83 PB were analyzed by high-resolution genome-wide molecular inversion probe analysis and methylation profiling. Seventy-nine cases were screened for mutations by next-generation DNA panel sequencing and for 25 samples mRNA expression was analyzed using NanoString. Additionally, 24 further pineal parenchymal tumors were analyzed. Clinical data of 63 patients was available. Our cohort consisted of 48 PB-miRNA1, 19 PB-miRNA2, 8 PB-MYC/FOXR2, and 8 PB-RB1 cases. PB-miRNA subtype tumors had characteristic alterations in microRNA-processing genes; *DICER1* mutations (n = 19/64) and homozygous deletions of the *DROSHA* locus (n = 18/67) were most abundant, followed by *DROSHA* mutations (n = 12/64). Most frequent cytogenetic aberrations in PB-miRNA cases were chromosome 7 gains (n = 31/67) and chromosome 14 losses (n = 26/67, including 5 cases with copy-neutral LOH). The latter were significantly associated with *DICER1* mutations (*p* < 0.001). *OTX2* gain represented the most frequent alteration that occurred in 37/83 PB of all subtypes. In the PB-miRNA subtypes we identified cases with polyploid cytogenetics (n = 16/67). In contrast to previous publications, we did not find a difference in survival for the PB-miRNA subtypes, whereas PB-MYC/FOXR2 and PB-RB1 in infants showed a worse outcome. Epigenetically defined PB subtypes are characterized by distinct genetic events. Frequent gains of the oncogene *OTX2* indicate a role in the pathogenesis of PB independent of its subtype.

**Supplementary Information:**

The online version contains supplementary material available at 10.1186/s40478-025-02140-7.

## Introduction

Pineoblastoma (PB) represents a rare, highly malignant pediatric supratentorial primitive neuroectodermal tumor arising in the pineal gland. Until 2020, only few data was available on the molecular and genetic background of this neoplasm. First, the presence of germline *RB1* mutations in a subset of PB in context of trilateral retinoblastomas indicated a role of this tumor suppressor gene. Indeed, alterations of the tumor suppressor gene *RB1* were also found in sporadic PB mostly occurring in infants [9, 27, 33]. In 2012, Sabbaghian et al. reported a germline *DICER1* mutation in a PB patient and the same group showed later that mutations in this gene predispose to PB in patients with *DICER1* syndrome [10, 11, 29]. Some years later homozygous deletions of *DROSHA*, another gene affecting the intracellular microRNA (miRNA) processing machinery acting upstream of *DICER1*, as well as microduplications in *PDE4DIP* were identified in PB [30]. In 2020, three research groups published data on non-overlapping cohorts of pineoblastoma (PB) and other pineal parenchymal tumors (pineal parenchymal tumors of intermediate differentiation (PPTID) and pineocytomas) showing that PB and PPTID consist of different molecular and clinical subtypes [18, 19, 27]. One year later a consensus paper with combined data of these cohorts was published further characterizing these subtypes [20]. PB subtypes (based on DNA methylation analysis) with alterations in miRNA-processing genes were defined as PB-miRNA1A, PB-miRNA1B, and PB-miRNA2. Whereas these miRNA-altered subtypes mainly affected older children and showed intermediate (miRNA1) or favorable (miRNA2) outcome, two other subtypes were primarily found in very young children with high incidence of tumor relapse despite intensive—though in infants mostly radiotherapy-sparing—treatment. The PB-MYC/FOXR2 subtype tumors presented recurrent gains or amplifications in the *MYC* gene and/or overexpression of *FOXR2*, an interaction partner of *MYC*. The tumors of the PB-RB1 subtype show alterations in the *RB1* gene and also recurrent gains in the miRNA-cluster mir-17/92 were described [20].

Whereas pineocytomas are benign pineal tumors with favorable survival but no known oncogenic driver alteration, PPTID (CNS WHO grade 2–3) show frequent inframe insertions in the *KBTBD4* gene as the main oncogenic driver [17, 20, 33]. Pfaff et al. described two subclusters of PPTID (PPTID-A and –B) [27].

To further investigate genomic alterations in these newly defined PB subtypes, we performed a systematic molecular, cytogenetic, and epigenetic analysis of an independent PB cohort. For those cases with clinical data available, our aim was to validate the favorable outcome of the miRNA-altered subtypes.

## Materials and methods

### Patients

From the archives of the Brain Tumor Reference Center of the German Society of Neuropathology and Neuroanatomy (DGNN) located in the Institute of Neuropathology in Bonn, Germany, a total of 147 cases diagnosed between 2000 and 2023 with a histological diagnosis of a pineal parenchymal tumor were identified. After neuropathological reevaluation and molecular analyses, eight cases were assigned to different tumor entities and removed from the cohort (4 AT/RT, 2 ETMR, 1 glioblastoma, 1 medulloblastoma). Further 32 cases with insufficient amounts or quality of DNA to precisely determine the molecular diagnosis with subtyping according to Liu et al. [20] were excluded (Supplementary Fig. 1). Sixty-three patients participated in the HIT-2000 trial (NCT00303810), the HIT-2000interim registry (NCT02238899), or the I-HIT-MED registry (NCT02417324) (clinical cohort). Informed consent had been given by the patients or their legal representatives. Five patients of this clinical cohort were part of the cohort published by Pfaff et al. and of the consensus cohort by Liu et al. (Supplementary Table 1) [20, 27].

### Histopathological and molecular analyses

According to the WHO classification 2021, the tumor samples were classified after conventional (HE and reticulin) and immunohistochemical stainings for synatophysin, OTX2, CRX (OTX3), and MIB-1 (Ki-67) [33]. DNA was extracted from fresh frozen tumor tissue (n = 5) or FFPE material (n = 101). In one case, both fresh frozen tumor and FFPE material was extracted and used for methylation array (fresh frozen) and molecular inversion probe array and NGS (FFPE).

### Molecular inversion probe array (MIP)

A MIP array including 330,000 inversion probes at single nucleotide polymorphism (SNP) sites (Version v2.0, Affymetrix, Santa Clara, CA, USA) was used as previously described to identify copy-number gains and losses [32]. Raw MIP data files were analyzed using the Nexus Copy Number 10.0 Discovery Edition software (BioDiscovery, El Segundo, CA, USA). SNP-FASST2-segmentation algorithm was used for copy number and loss of heterozygosity calling after diploid correction. When at least 90% of the probe signals of a chromosome were above/below the defined thresholds, these chromosomes were counted as whole chr gains/losses. For acrocentric chromosomes only q-arms were analyzed. Sex chromosomes were excluded from the determination of the numerical extent of whole chromosomal aberrations (WCA) [13]. Polyploidy was defined as > 15 whole chromosomal gains and the absence of copy losses. Genomic Identification of Significant Targets in Cancer (GISTIC) analysis was used to identify significant focal chromosomal aberrations (p-level: 0.005) [4].

### Next-generation sequencing (NGS)

103 tumor samples were assessed using Illumina DNA Prep for Enrichment (Nextera Flex) NGS panel (Illumina, San Diego, CA, USA). Libraries were generated from 100 to 400 ng FFPE DNA with ~ 9000 custom enrichment probes covering the coding regions and exon–intron boundaries of 89 genes or their mutational hotspots frequently mutated in medulloblastoma and pineoblastoma (Supplementary Table 2). Purified and normalized libraries were pooled and subsequently sequenced on a MiSeq System (Illumina) with reagent kit v2 (TSCA). Alignment of reads, variant calling, and annotation was performed with BWA using the Illumina MiSeq Reporter and Variant Studio v3.0 software. Variants were manually curated using the Variant Studio v3.0 software. BAM files were uploaded in the Nexus Copy Number 10.0 software for analysis of copy number alterations in the 89 genes.

### Genome-wide DNA methylation profiling

Fresh-frozen or FFPE-derived DNA was used on the Illumina Infinium HumanMethylation450 (450k; n = 2) or EPIC BeadChips (850k; all others). Data processing was performed as previously outlined [8, 15, 27]. The Heidelberg Brain Tumor classifier v12.5 was used to annotate the profiles to a methylation class. Scores > 0.9 were considered as match, but samples with lower scores were also part of the final cohort when they unequivocally clustered with other samples of the same subtype. For this, DNA methylation profiles from all samples were compared to the meta-analysis cohort of Liu et al. (n = 216) [20] and a combined CNS tumor reference cohort from Capper et al. and Liu et al. (n = 2976 together) [8, 20] using t-stochastic neighbor embedding (t-SNE) visualization [23]. Also, uniform manifold approximation and projection (UMAP) plotting was performed using the EpiDiP platform [14, 24]. Derived copy number variation profiles (CNVP) were compared to those from MIP array to validate copy number alterations and exclude sample mismatches.

### Messenger-RNA (mRNA) expression analysis

FFPE-derived RNA was analyzed on the nCounter NanoString platform (NanoString Technologies, Inc. (Seattle, WA, USA)). RNA was isolated with Qiagen’s miRNeasy FFPE Kit. mRNA expression was measured with nCounter® Tumor Signaling 360™ Panel (NanoString Technologies, Inc.). Statistical analysis was perfomed with Rosalind Software, ROSALIND, INC. (San Diego, CA, USA), v3.38.09.0 and NanoString’s nSolver Software, v4.0. P-values were adjusted with the Benjamini–Hochberg method. Plots were created using IBM SPSS Statistics (v29).

### Statistical analysis

Clinical risk factors were evaluated using univariable log-rank tests for progression-free survival (PFS) and overall survival (OS), with results reported via the Kaplan–Meier method as estimates ± standard errors. Age was modeled as a categorical variable, with a cut-off of 4 years at diagnosis used to distinguish between younger (< 4 years) and older (≥ 4 years) patients. This cut-off was historically utilized in HIT-affiliated studies as a parameter for determining the temporal sequence of radiotherapy and chemotherapy to reduce neurocognitive side effects from radiotherapy. PFS and OS were defined as the interval from the date of the first resection or biopsy to the date of first progression/relapse/death or death, respectively, with survival data censored at the time of the last evaluation. Statistical analyses and graphical representations were performed using IBM SPSS Statistics (version 27) and R version 4.2.0.

## Results

**Fig. 1:**
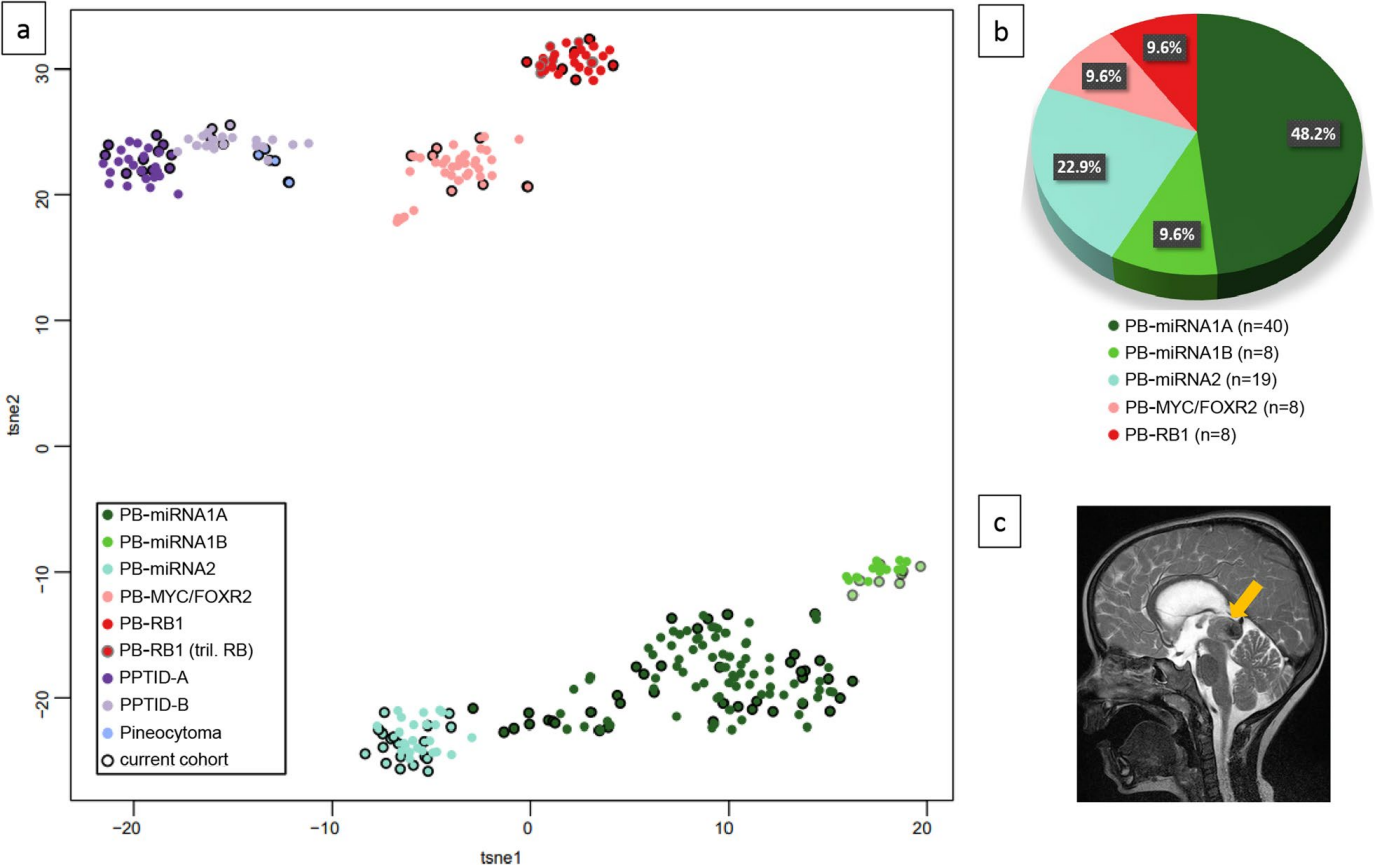
**a** t-SNE plot of 104 samples from the current cohort (the two pineal anlage tumors and the ectopic WNT-MB were omitted) together with the consensus cohort from Liu et al. (n = 216; an overlap of 5 cases from the current cohort and the Liu cohort exists (3 PB-miRNA1A, 1 PB-miRNA1B, 1 PPTID-B)); the trilateral PB-RB1 cases from the current cohort are shown with a big grey frame (n = 2), those from the Liu cohort with a small grey frame (n = 5). **b** Pie chart showing the distribution of the PB subtypes in the current cohort. **c** MRI showing a representative lesion in the pineal region from a patient with a PB-miRNA1 subtype tumor (arrow)

In the final cohort of 107 pineal parenchymal tumors 104 were defined based on methylation profiling and subsequent t-SNE analysis. We identified 83 PBs, 15 PPTIDs, and 6 pineocytomas (Fig. 1a and Supplementary Fig. 2a-c). Within the PBs, 40 tumors were of the PB-miRNA1A subtype, whereas PB-miRNA1B tumors accounted for only 8 cases. Nineteen cases belonged to the PB-miRNA2 subtype. In addition, 8 cases represented PB-MYC/FOXR2 and 8 samples PB-RB1 subtypes (Table 1 and Fig. 1b). Furthermore, two pineal anlage tumors (PAT) and one WNT-activated PB arising from the pineal gland were diagnosed by histopathological and molecular genetic findings other than methylation.

**Table 1:** Cohort overview—Demographical and clinical characteristics

		Whole PB cohort			PB-miRNA1A			PB-miRNA1B			PB-miRNA2			PB-MYC/FOXR2			PB-RB1		
		All n = 83	Clinical n = 55	Comparison *p-*value*	All n = 40	Clinical n = 27	Comparison *p-*value*	All n = 8	Clinical n = 5	Comparison *p-*value*	All n = 19	Clinical n = 13	Comparison *p-*value*	All n = 8	Clinical n = 5	Comparison *p-*value*	All n = 8	Clinical n = 5	Comparison *p-*value*
**Sex**																			
Male		46 (55%)	32 (58%)	*p* = 0.48	12 (30%)	8 (30%)	*p* = 0.94	5 (63%)	3 (60%)	*p* = 0.85	16 (84%)	11 (85%)	*p* = 0.94	6 (75%)	5 (100%)	*p* = 0.04	7 (88%)	5 (100%)	*p* = 0.17
Female		37 (45%)	23 (42%)		28 (70%)	19 (70%)		3 (37%)	2 (40%)		3 (16%)	2 (15%)		2 (25%)	0 (0%)		1 (12%)	0 (0%)	
Male:female ratio		1.2:1	1.4:1		1:2.3	1:2.4		1.7:1	1.5:1		5.3:1	5.5:1		3:1	–		7:1	–	
**Mean age at diagnosis in years (range)**		11.8 (0.2–54.0)	11.0 (0.2–30.8)	–	12.8 (2.1–49.0)	11.7 (2.1–30.8)	–	12.6 (4.0–31.0)	12.0 (4.0–24.4)	–	17.2 (8.0–54.0)	15.7 (8.8–25.6)	–	2.1 (0.8–4.0)	1.8 (0.8–3.6)	–	2.5 (0.2–4.6)	3.1 (0.2–4.6)	–
**M-status and resection status**																			
M0R0 (< 1.5cm2)		–	15 (27%)	–	–	10 (37%)	–	–	0	–	–	2 (15%)	–	–	1 (20%)	–	–	2 (40%)	–
M0R +		–	15 (27%)		–	6 (22%)		–	2 (40%)		–	5 (39%)		–	2 (40)%		–	0	
M +		–	25 (46%)		–	11 (41%)		–	3 (60%)		–	6 (46%)		–	2 (40%)		–	3 (60%)	
**Radiotherapy (as first-line therapy)**																			
Infants		–	3 (of 9)	–	–	1 (of 1)	–	–	0 (of 0)	–	–	0 (of 0)	–	–	1 (of 5)	–	–	1 (of 3)	–
Non-infants		–	46 (of 46)		–	26		–	5 (of 5)		–	13 (of 13)		–	0 (of 0)		–	2 (of 2)	
**Survival status**																			
No event		–	30 (55%)	–	–	17 (63%)	–	–	3 (60%)	–	–	8 (62%)	–	–	1 (20%)	–	–	1 (20%)	–
PFS: Relapse/ Progression/Death		–	25 (45%)		–	10 (37%)		–	2 (40%)		–	5 (38%)		–	4 (80%)		–	4 (80%)	
OS: Death		–	22 (40%)		–	9 (33%)		–	2 (40%)		–	5 (38%)		–	3 (60%)		–	3 (60%)	
**Follow-up (in years)**																			
Median (range)		–	4.06 (0.26 – 19.49)	–	–	3.89 (0.26 – 18.28)	–	–	4.77 (2.77 – 19.49)	–	–	4.49 (2.22 – 17.22)	–	–	2.39 (0.74 – 18.91)	–	–	2.09 (1.17 – 7.75)	–

^*^ Clinical cohort cases with PFS/OS versus remaining cases compared using chi^2^-test

### Characteristics of PB-miRNA subtypes

**Fig. 2:**
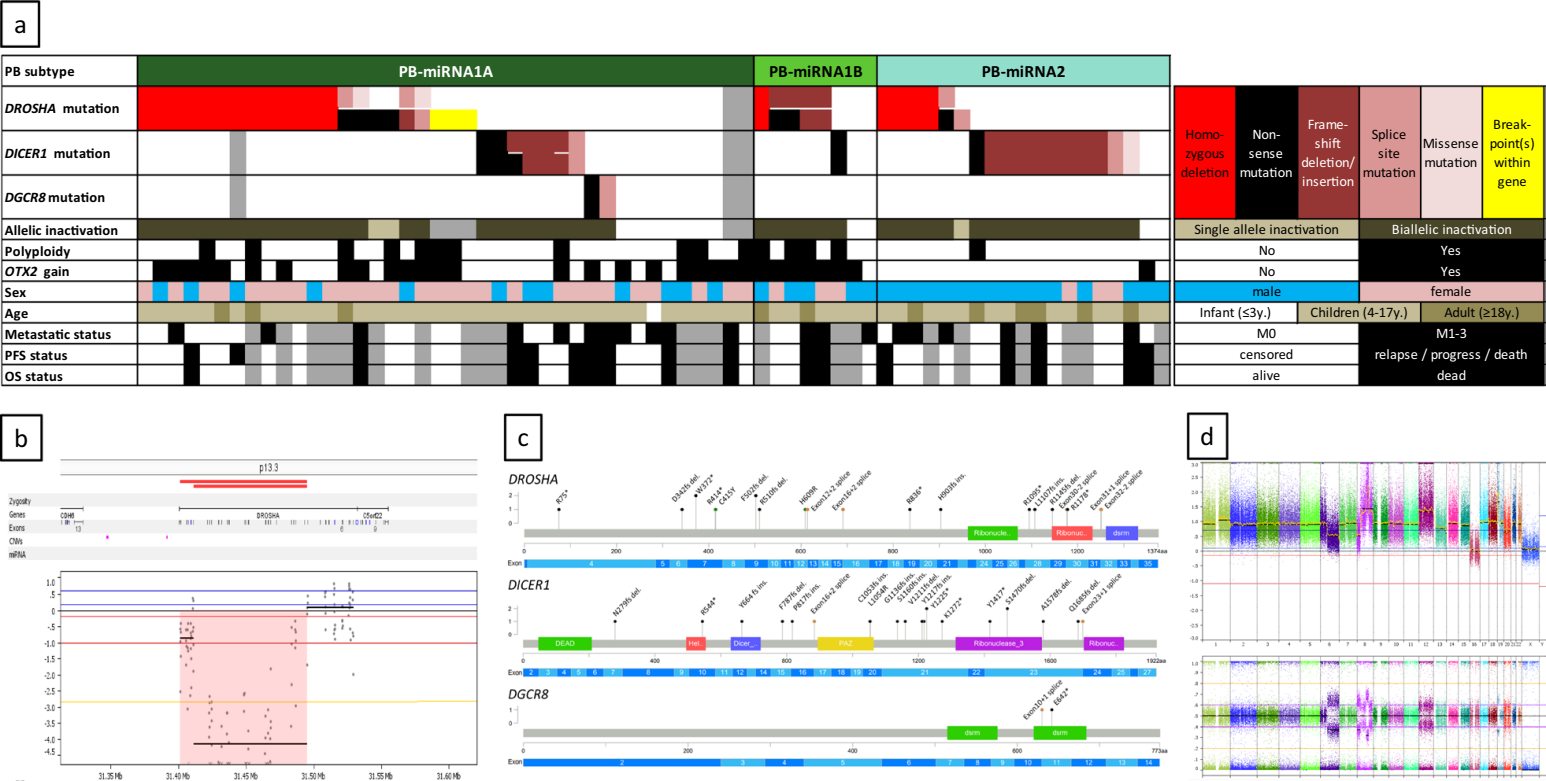
**a** Overview of the 67 miRNA-altered pineoblastoma (PB) subtypes (PB-miRNA1A, n = 40; PB-miRNA1B, n = 8; PB-miRNA2, n = 19). Missing data are shown in grey. **b** Copy number profile of the *DROSHA* locus from a PB-miRNA1A tumor sample with a partial homozygous deletion of the *DROSHA* gene. **c** Overview of the variants in the miRNA-processing genes *DROSHA*, *DICER1*, and *DGCR8*. **d** Genome-wide copy number plot from a PB-miRNA1B tumor with a polyploid genome. Abbreviations: mut., mutation; Chr., chromosome; LOH, loss of heterozygosity; m, mosaic; cn, copy-neutral; cg, copy gain; y., years; PFS, progression-free survival; OS, overall survival; dsrm, double-stranded RNA binding motif; PAZ, Piwi-Argonaute-Zwille

Patients with PB-miRNA1A subtype tumors were predominantly female, whereas those with PB-miRNA1B and PB-miRNA2 tumors were predominantly male. Patients with PB-miRNA1A and -miRNA1B subtype tumors were mostly in childhood age with only one infant in these subtypes and significantly younger than patients with PB-miRNA2 tumors (p = 0.033; t-test; Table 1). Mutations in the previously published miRNA-processing genes were found in the majority of cases (54 of 65, 83%; no data available for 2 cases). Whereas the two mutations in *DGCR8* were both in PB-miRNA1A tumors, alterations in *DICER1* (n = 19) and *DROSHA* (n = 33) were present in all three PB-miRNA subtypes (Fig. 2a and Supplementary Fig. 3). The mutations in the different miRNA-processing genes were mutually exclusive. The mutations represented biallelic inactivation, with the exception of six cases carrying DROSHA mutation: three cases showed chromosomal breakpoints within the gene (Supplementary Fig. 4a-c) and three other cases had only one mutated allele (Fig. 2a). Biallelic inactivations of *DROSHA*, *DICER1*, or *DGCR8* occurred with either two mutations with VAF of ~ 30–50% each (compound heterozygous), or one mutation with VAF of ~ 75–100% and a copy loss at the gene locus. Homozygous deletions of *DROSHA* found in 18 PB samples mostly affected the whole gene, but in some cases only partial deletions of *DROSHA* were observed (Fig. 2b). Most mutations were truncating, whereas missense mutations were very rare (n = 3). *DROSHA* and *DICER1* mutations were found all over the gene with only very few recurrent mutations (Fig. 2a and c). In further 11 cases (17%) with sequencing data available no mutation in one of these genes of the miRNA-processing pathway was found, but we detected a homozygous deletion of the *BCOR* locus in one case as well as missense variants in *GFI1* (VAF = 72%), *GSE1* (VAF = 38%), and *ELP1* (VAF = 25%) in one case each (not shown). Further *BCOR* variants were found in a PB-miRNA2 case with a homozygous *DROSHA* deletion (*BCOR* S17R; VAF = 100%, uncertain significance of pathogenity) and in a PB-miRNA1A tumor with a heterozygous *DROSHA* mutation (*BCOR* P120 frameshift deletion; VAF = 18%). Of note, as for *BCOR*, variants in *GFI1* and *GSE1* were also found in single tumor samples with mutations in miRNA-processing genes (not shown).

**Fig. 3:**
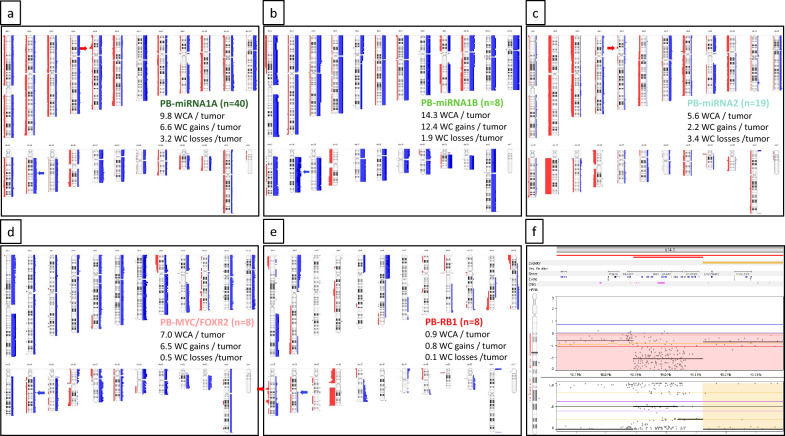
**a**-**e** Summary plots of copy number aberrations from molecular inversion probe (MIP) array from pineoblastoma (PB) subtypes. Blue bars, gains; red bars, losses. Thickness of bars indicates frequency of alterations; red arrows show the *DROSHA* locus (**a** and **c**) and the *RB1* locus (**e**), blue arrows show the *OTX2* locus (**a**, **b**, **d**, **e**). WCA, whole chromosomal aberrations. Of note, WC losses include copy-neutral losses of heterozygosity, but these are not visible in the summary plots. (**f**) Copy number profile of the *RB1* locus from a PB-RB1 tumor sample with a focal homozygous deletion of the *RB1* gene (see also Supplementary Fig. 6b-c)

Cytogenetic analysis revealed a total of 16 tumors with a polyploid phenotype (Fig. 2a and d). These were especially enriched in PB-miRNA1B tumors (4 of 8), but also in PB-miRNA1A tumors (11 of 40). In PB-miRNA2 tumors only one polyploid case was found. Almost all PB-miRNA2 cases had a whole chromosome 14 loss (17 of 19; *p* < 0.001 (compared to PB-miRNA1A/B)). PB-miRNA2 cases showed fewer whole chromosomal aberrations (5.6 WCA) in contrast to the PB-miRNA1A tumors with a mean of 9.8 WCA, and the PB-miRNA1B with a mean of 14.3 WCA. In a genome-wide comparison of frequency of whole genomic alterations between PB-miRNA1A and 1B therefore several whole chromosomal gains were significantly enriched in subtype 1B, whereas the focal (homozygous) *DROSHA* deletions were significantly more frequent in subtype 1A (not shown). In total, chromosome 14 losses were found in 38.8% of PB-miRNA tumors (26/67, including 5 cases with copy-neutral loss of heterozygosity (cnLOH)). Even more frequent, whole chromosome 7 gains occurred in 46.3% tumors (31/67) and chromosome 12 gains in 41.8% (28/67); Fig. 3a-c and Supplementary Fig. 3). *DICER1* mutations were significantly associated with chromosome 14 loss (*p* < 0.001) and subtype PB-miRNA2 (p = 0.002). Twenty of the PB-miRNA cases showed chromosome 14 gains, and 11 additional cases single copy gains of the *OTX2* oncogene region on chromosome 14q22.3 only, of those 8 focal and 3 large gains. In GISTIC analysis, copy number alterations of the *DROSHA* and the *OTX2* loci represented significant alterations (Supplementary Fig. 4d). Amplifications were only found in 4 tumor samples, mainly as larger amplified regions in polyploid cases (Fig. 2d and Supplementary Figs. 4a and 5a). Only one non-polyploid tumor had a focal amplification at chromosome 19q including several zinc finger protein encoding genes (not shown). Of note, the amplified regions at chromosome 5p in the example in Supplementary Figs. 4a and 5a were part of a chromothryptic chromosomal area. Chromothrypsis was also detected in two other cases and affected chromosome 10 and chromosome arm 15q, respectively (Supplementary Fig. 5b and c).

### PB-MYC/FOXR2 and PB-RB1

The non-miRNA-altered PB subtype patients were significantly younger than those with PB-miRNA-altered tumors and showed a strong male predominance in our cohort. Patients with PB-MYC/FOXR2 subtype tumors had a mean age of 2.1 years and those with PB-RB1 tumors of 2.5 years (Table 1). Subtyping based on methylation profiling for the PB-MYC/FOXR2 tumors appeared difficult for several cases as in 6 tumors no clearly matching score in the Heidelberg classifier v12.5 were found and in 5 of these cases the highest available score was even for subtype III medulloblastoma (not shown). However, as these tumors all located in the pineal gland and all 8 cases unequivocally clustered together and with previously published tumors of this subtype in a subsequent t-SNE-analysis we included these cases in our cohort (Fig. 1a). Two of the patients with PB-RB1 had a history of retinoblastoma and were therefore diagnosed as trilateral retinoblastoma. By NGS panel sequencing and MIP array we could not detect any pathogenic or likely pathogenic variants in PB-MYC/FOXR2 tumors, whereas most PB-RB1 tumors showed biallelic inactivation of the *RB1* gene at chromosome 13q14.2. In three cases focal homozygous deletions of the *RB1* locus were found and three other cases had truncating *RB1* mutations with VAF of 85–100% and additional copy-neutral/copy-gain LOH. The remaining cases also both had LOH of the *RB1* locus (one a slightly mosaic cnLOH and the other one a copy loss), but from the first case NGS analysis was not possible and the other did not show a *RB1* mutation (Fig. 3e and f and Supplementary Fig. 6a-d). Only in one tumor we found an additional pathogenic variant with our NGS panel, which was a truncating *BCOR* mutation (R1472 frameshift-deletion; VAF of 80%) in a PB-RB1 tumor with a homozygous *RB1* deletion (not shown).

As in the PB-miRNA tumors also in two of the PB-MYC/FOXR2 tumors we found a polyploid genome. Of note, in both these tumor genomes chromosome 16 did not show a gain but was balanced or showed a cnLOH, respectively (Fig. 3d and Supplementary Fig. 6a), and two further cases had whole chromosome 16 or chromosome arm 16q losses. Three non-polyploid tumors had recurrent losses of chromosome arm 8p and gains of 8q including the *MYC* region. However, amplifications of *MYC* or any other gene were not detected. Two of the PB-MYC/FOXR2 tumors showed an isochromosome 17q (i17q). In the PB-RB1 tumors WCA were rarely seen except for chromosome 16 losses (3 of 8); 3 further cases had chromosome arm 16q losses. In addition to alterations of the *RB1* locus several recurrent focal regions or chromosome arms were altered like (partial) chromosome 1q gains, 2p gains, 6p gains, 12p losses, and 17q gains (Fig. 3e). Of note, all four PB-RB1 tumors harboring deletions of the *RB1* locus also showed losses of a second region on chromosome arm 13q31.1 including genes like *RBM26* or *NDFIP2*. The previously described gains in the *miR17/92* cluster, also located on chromosome arm 13q, were found in three cases, even though an amplification of this locus was not identified (Supplementary Fig. 6c). GISTIC analyses in these two subtypes revealed no significant focal regions other than the *RB1* locus in the PB-RB1 tumors (not shown). Also the *OTX2* locus was not detected as significant aberration by GISTIC in both non-miRNA-altered subtypes, but similar to PB-miRNA tumor focal *OTX2* and whole chromosome 14 gains (including *OTX2*) were present in PB-MYC/FOXR2 (n = 4) and PB-RB1 cases (n = 2; Fig. 3d and e and Supplementary Fig. 6a). Therefore, tumors with *OTX2* gain were present in all PB subtypes.

### Non-PB tumors

Both PAT patients were young infants and the tumors had typical histological characteristics with undifferentiated components, as well as neuronal, melanotic, and mesenchymal phenotypes. Cytogenetic analysis revealed mostly stable genomes, but both tumors had identical losses at chromosome arm 8p and gains at chromosome arm 17q. Both PAT had high scores for PB-MYC/FOXR2 based on DNA methylation profiling. Pathogenic or likely pathogenic variants were not detected in the genes covered by our NGS panel. The ectopic WNT medulloblastoma (PB with WNT activation) showed nuclear β-catenin accumulation in only a small subfraction of tumor cells (< 5%), but a mosaic monosomy 6 and an activating *CTNNB1* mutation. Of note, this case was clearly located in the pineal region without contact to the cerebellum and also showed a truncating *DROSHA* mutation. The WNT-activated PB had a methylation signature of WNT medulloblastomas (score 0.78 in v12.5 of the Heidelberg classifier) and clustered with these in t-SNE plots (not shown). Detailed information on these three cases can be found in Supplementary Figs. 7 and 8.

The 15 PPTID were mainly of subtype PPTID-A (n = 11; 6 male and 5 female; mean age 34.2 years (range 5–72 years)). The 4 PPTID-B were all female and had a mean age of 40.9 years (range 17–68 years). The WHO grading revealed mainly grade 3 (11 of 15, 73%). All PPTID and pineocytomas had mostly stable genomes with only few focal or numerical alterations. Whereas the pineocytomas showed no pathogenic variants in NGS panel sequencing except for a heterozygous *PALB2* variant in a single case, hotspot *KBTBD4* inframe insertions were found in all 4 PPTID-B samples and 9 of 11 PPTID-A cases. Summary plots of MIP arrays and further detailed information can be found in Supplementary Fig. 9.

### mRNA expression analysis

**Fig. 4:**
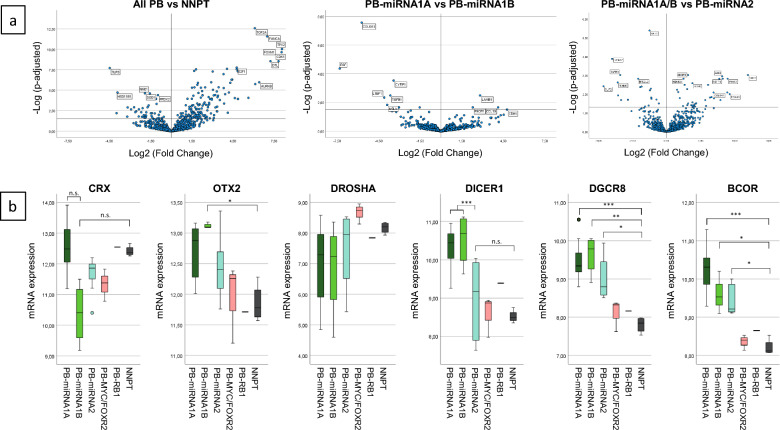
**a** Volcano plots from mRNA expression analyses showing differential expression between tumors from all analyzed pineoblastoma (PB) samples (n = 25) versus non-neoplastic pineal tissue (NNPT) samples (n = 4; left), PB-miRNA1A (n = 9) versus PB-miRNA1B (n = 4; middle), and all PB-miRNA1 (n = 13) versus PB-miRNA2 (n = 8; right). **b** Comparison boxplots of expression levels of *CRX*, *OTX2*, *DROSHA*, *DICER1*, *DGCR8*, and *BCOR* between PB tumor subtypes and NNPT

Expression levels of 746 mRNAs were analyzed in a series of 9 PB-miRNA1A, 4 PB-miRNA1B, 8 PB-miRNA2, 3 PB-MYC/FOXR2, and 1 PB-RB1. As control we used 4 non-neoplastic pineal tissue (NNPT) samples from pineal cyst walls. In a comparison of all 25 PB samples against the NNPT mainly cell cycle-regulating genes like *TOP2A*, *CDK1*, *FANCA*, or *AURKB* were overexpressed in PB tumors (Fig. 4a). When comparing the two PB-miRNA1 subtypes, PB-miRNA1A showed a higher mRNA expression of *LAMB1*, *PIK3R1*, *CCL19*, and *CDH1*. In contrast, overexpressed in PB-miRNA1B tumors were *COL6A1* and *EGF* (Fig. 4b) with *COL6A1* also significantly overexpressed in all PB-miRNA1 tumors when compared to PB-miRNA2 tumors, along with *DICER1* or *FGFR1*. In this comparison, *AKT2* and *LAMA2* were amongst the most overexpressed mRNAs in PB-miRNA2 tumors (Fig. 4a).

The pineal lineage marker *CRX* (*OTX3*) and the oncogene *OTX2* were both highly expressed in all PB subtypes and among the top most expressed genes overall. *DGCR8* was significantly higher expressed in all three PB-miRNA subtypes. For *DICER1*, a significant higher mRNA expression was found in PB-miRNA1A and -miRNA1B tumors only, but not in the PB-miRNA2 subtype. Finally we analyzed the expression levels of *BCOR* in the PB subtypes as this gene was found mutated in several tumors. Indeed, expression of *BCOR* was significantly higher in all PB-miRNA subtype tumors than in NNPT, but not in the PB-MYC/FOXR2 or PB-RB1 tumors (Fig. 4b).

### Survival analysis

Clinical data were available for 55 PB patients, 5 PPTID, 1 pineocytoma, 1 PAT, and the patient with the WNT-activated PB (ectopic WNT-medulloblastoma).

**Fig. 5:**
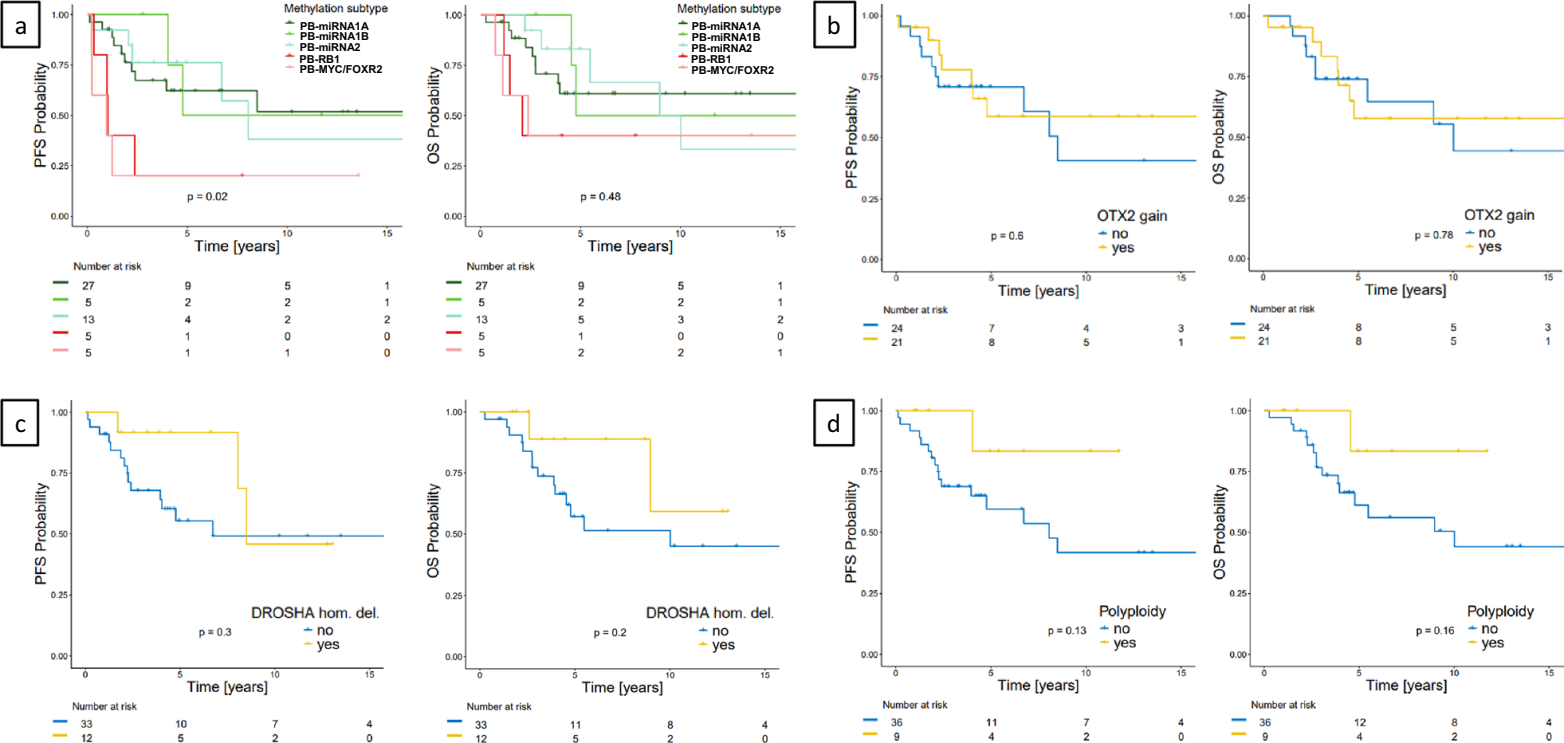
Kaplan–Meier PFS and OS plots for **a** pineoblastoma (PB) methylation subtypes (n = 55 with clinical data), **b** *OTX2* gains (focal and whole chromosome 14 combined) in all 45 PB-miRNA subtype tumors with clinical data, **c** PB-miRNA subtype tumors with homozygous *DROSHA* deletions, and **d** PB-miRNA subtype tumors with polyploid genome

Patients with different PB-miRNA subtype tumors showed similar progression-free and overall survival with intermediate to poor outcome and 5-year PFS/OS of 62.1 ± 10.1%/60.8 ± 10.3% (PB-miRNA1A; n = 27), 50.0 ± 25.0%/50.0 ± 25.0% (PB-miRNA1B; n = 5), and 76.2 ± 12.1%/83.1 ± 11.0% (PB-miRNA2; n = 13). A detailed overview with clinical information of all PB-miRNA2 patients is shown in Supplementary Table 3. Patients with PB-MYC/FOXR2 and PB-RB1 tumors (n = 5 each with clinical data) had significantly worse PFS than those with PB-miRNA tumors with 5-year PFS of 20.0 ± 17.9% (both PB-MYC/FOXR2 and PB-RB1). However, OS was not significantly worse as in both subtypes two of the five patients each were long-term survivors, even though one of them each had a tumor relapse. The 5-year OS for patients with PB-MYC/FOXR2 and PB-RB1 was both 40.0 ± 21.9% (Figs. 2a and 5a; Supplementary Figs. 3 and 6a). Of note, all but one relapsing patient with a PB-miRNA subtype tumor died.

When analyzing the patients with all three PB-miRNA subtype tumors combined regarding a possible prognostic role of molecular markers like whole chromosome 7 gains or whole chromosome 14 losses, no differences in PFS or OS was found (not shown). Also *OTX2* gains (focal, large, and whole chromosome 14 combined) and homozygous *DROSHA* deletions had no prognostic impact (Fig. 5b and c). One marker showed a trend towards improved outcome, which was the highly polyploid phenotype (Fig. 5d).

The 5 PPTID (4 PPTID-A and 1 PPTID-B) and the pineocytoma patient were all alive after last follow-up (median follow-up: 5.57 years without tumor relapse, even though all 5 PPTID showed high proliferation rates and were assigned to WHO grade 3 (Supplementary Fig. 9). The PAT patient had an early tumor relapse and died approximately 1 year after the relapse, whereas the patient with the WNT-activated PB was alive and tumor-free at last follow-up 13.8 years after diagnosis (not shown).

## Discussion

### Pineal parenchymal tumors represent a group of biologically and (epi)genetically distinct tumor types

Several different tumor types are known to originate in the pineal gland including PB, PPTID, pineocytomas or papillary tumors of the pineal region (PTPR). Recent research led to the discovery of further molecular subtypes especially in PB. Around 65–80% of PB belong to the miRNA-altered subtypes. Validating the data presented by Liu et al. [20] we also showed the different age distribution with mostly older patients in the miRNA-altered PB subtypes and a predominance of infant patients in the PB-MYC/FOXR2 and PB-RB1 subtypes. We also validated the common and subtype specific molecular and cytogenetic alterations between the two PB-miRNA1 subtypes (namely 1A and 1B) and the PB-miRNA2 tumors, with chromosome 14 losses and *DICER1* mutations significantly enriched in PB-miRNA2 tumors. This also had consequences visible in mRNA expression, as *DICER1* expression was significantly reduced in PB-miRNA2 tumors, which was not shown by Liu et al. before. In the consensus paper by Liu et al. PB-miRNA subtypes 1A and 1B were combined due to a lack of significant clinical and biological differences [20]. And except for a higher percentage of polyploidy in PB-miRNA1B tumors and predominantly male patients with this tumor subtype, which were both not significant when compared to (patients with) PB-miRNA1A tumors, also in our cohort we did not find arguments for separating these two epigenetic subtypes in future studies. Moreover, in UMAP visualization, clusters of these subtypes were partly overlapping making even an epigenetic distinction difficult.

### Mutational spectrum of PB subtypes

Most of the PB-miRNA tumors harbored biallelic mutations in miRNA-processing genes but 17% of cases lacked mutations in these driver genes. Alternative genetic events may drive these tumors. However, other genes involved in the miRNA-processing machinery like *XPO5* and *TARBP2* were not part of our NGS panel. These were screened for mutations in the consensus cohort of Liu et al. [20], but no mutations were reported. A single PB with a biallelic inactivation of the *PBRM1* gene—a tumor suppressor which appears to drive cell growth and genomic instability, but not part of the miRNA processing machinery—and chromosome 14 loss was recently published by Antonios et al., likely showing an alternative tumor-initiating event [1]. Liu et al. found *DROSHA/DICER1/DGCR8*-wildtype tumors in 35% (29 of 82) of PB-miRNA tumors, compared to 17% presented here (11 of 65) [20]. This discrepancy may be related to the frequency of detected *DROSHA* alterations; a careful screen especially of the *DROSHA* locus for intra-genomic breakpoints and small (homozygous) deletions is recommended, as in some cases alterations were only detected by our NGS analysis, but hardly visible in copy-number plots derived from MIP or methylation arrays. Further analysis of PB samples by whole genome sequencing (WGS) may identify alternative tumorigenic driver genes. As a hint for recurrently altered genes so far undescribed in PB, chromosomal alterations may accumulate in loci including such genes. However, in a comparison of high-resolution copy number data (MIP array) with the 11 *DROSHA/DICER1/DGCR8*-wildtype PB-miRNA samples of our cohort against the 54 mutant tumors no significant cytogenetic differences were found (not shown), and Liu et al. found no survival differences between these two groups in their cohort [20].

*PDE4DIP* microduplications were identified by Snuderl et al. in 7 of 15 analyzed PB by WGS and confirmatory digital droplet PCR, but these were not visible in CNVPs of methylation arrays [30]. However, such microduplications were not described in any other PB publication; also in our cohort we were unable to detect them by high-resolution MIP array [18–20, 27]. One explanation for this discrepancy is that these duplications are too small and may not be densely covered by MIP probes. It also remains unclear in which subtype(s) Snuderl et al. detected this alteration.

A novel finding of our mutational analysis was the identification of recurrent variants in the *BCOR* (*BCL6 corepressor*) gene (n = 4), which is a gene involved in embryogenesis and implicated in transcriptional regulation by mediating transcriptional repression through histone modifications [2]. Of note, also in other embryonal tumors like retinoblastomas or medulloblastomas as well as in glial tumors mainly truncating loss-of-function *BCOR* mutations were reported [2, 16, 25, 26]. However, the exact biological impact of these mutations is largely unresolved. The significantly increased mRNA expression of *BCOR* was shown before by Trubicka et al. in a small series of PB. On the protein level all analyzed tumor samples of this study showed nuclear staining of BCOR, but internal tandem duplications in exon 15 of the *BCOR* gene or *BCOR* gene fusions were absent [31]. Even though the pathogenic impact of the *BCOR* variants presented in our cohort remains unclear, the data presented here and by Trubicka et al. indicate a possible role of *BCOR* in PB, at least in miRNA-altered subtypes.

In PPTID *KBTBD4* mutations as tumor initiating event were found, but *KBTBD4*-wildtype tumors were also identified. Liu et al. described that adult patients with PPTID were more likely to have *KBTBD4*-mutant tumors than younger patients [20]. However, in the current cohort the only two *KBTBD4*-wildtype tumors were both observed in older adults, whereas all six tumors from patients younger than 18 years were *KBTBD4* mutant, although the sample size was too small for a statistical comparison. In the analyzed pineocytoma and PAT samples no mutations were found except for a *PALB2* mutation in one pineocytoma. The WNT-activated PB harbored a truncating *DROSHA* mutation characteristic for miRNA-altered PB and a *CTNNB1* mutation typically seen in WNT-activated medulloblastomas. As Liu et al. published a series of 7 such tumors with mostly methylation signatures of WNT-activated medulloblastomas and *CTNNB1* mutations, but additional variants in miRNA-processing genes were not described [21], it would be interesting to sequence such tumors more carefully in the future to find out if more (or all) of these cases share features of both miRNA-altered PB and WNT medulloblastomas. However, the detection of a characteristic PB-related alteration in our case argues against the term “ectopic WNT medulloblastoma” but strengthen the hypothesis that such tumors represent a unique PB subtype.

### PB-miRNA-altered subtypes did not show differences in clinical outcome

Survival analyses showed that the mostly infant patients with both non-miRNA-altered PB subtype tumors presented worse outcome compared to those with PB-miRNA1 and -miRNA2 tumors. Even though in OS only a trend was visible—most likely because of low numbers—this validated the results by Liu et al. [20]. In older patients with miRNA-altered PB the 5-year PFS/OS is 50–76%/50–83%, and no difference was found between patients with PB-miRNA1 and -miRNA2 tumors, whereas Liu et al. showed a very favorable outcome for PB-miRNA2 patients. Whether this finding may be related to different treatment modalities (degree of resection, exact dose of chemotherapy and craniospinal irradiation (CSI)) remains elusive. Our data as well as those from the published consensus cohort represent retrospective analyses with restrictive value of definitive conclusions on survival. So far, our finding argue against the consideration of reduced treatment intensity for PB-miRNA2 patients without previous validation in prospective studies.

Whereas relapses in patients with PB-miRNA tumors often occur several years after initial diagnosis, tumor relapses in patients with PB-MYC/FOXR2 and PB-RB1 mostly occur rapidly after primary surgery. Given the usually very young age at diagnosis patients might therefore still not have reached an age at which radiotherapy is safe to administer. Further studies will be required to investigate methods to either delay radiotherapy in front-line therapies or to make therapy more effective. To this end, the identification of positive prognostic markers from long-term survivors would help to develop more tailored treatment regimen and therefore to avoid treatment-related sequelae after aggressive therapy. In general it remains unclear if omitting upfront radiotherapy due to the younger age in patients with PB-MYC/FOXR2 and PB-RB1 tumors alone is responsible for the worse outcome, or if the tumor biology of these two subtypes has a prognostic impact and leads to the inferior survival compared to patients with PB-miRNA tumors.

### Polyploid karyotypes in PB

We newly identified a cytogenetic phenotype of polyploidy with whole gains in the majority of chromosomes occurring in all PB subtypes except for PB-RB1. A superior outcome of patients with tumors showing whole chromosomal aberrations is known e.g. for medulloblastoma [13], but also for patients with the whole-chromosomal changes phenotype in neuroblastoma and high hyperdiploid acute lymphoblastic leukaemia. However, polyploidy was not significantly associated to survival, but a trend towards improved PFS and OS was observed (Fig. 5d). Of note, tumors with highly polyploid genomes can only be found by WGS or SNP-based arrays like MIP, but even with these methods polyploid tumors could be missed if not single chromosomes were found that could be used for a diploid correction. Therefore, it is possible that even more tumors of our cohort harbor such a phenotype. A validation of this trend presented here or the identification of possible other cytogenetic prognostic markers needs further examination in larger cohorts and preferably prospective studies.

### OTX2 gains and overexpression suggests an oncogenic role in the pathogenesis of PB

*OTX2* (orthodenticle homeobox 2) gains—another newly identified cytogenetic event in PB—occured in 45% of PB and in all subtypes. On the chromosomal level, these gains were of different extension ranging from whole chromosomal gain to focal single gene gain. In fact, *OTX2* gain represented the second most frequent focal alteration in our PB cohort after *DROSHA* deletions. Although this alteration had no prognostic impact, the finding suggests a pathogenic role of this transcription factor in PB. For medulloblastoma, another embryonal tumor originating in the cerebellum, an oncogenic role with overexpression of *OTX2* was shown mainly in non-WNT/non-SHH medulloblastomas [6, 7, 22]. We found that focal gains also in WNT-activated medulloblastomas were related to poor prognosis [12]. Even germline duplications of chromosome 14q22.3 including *OTX2* were described rarely in medulloblastomas [5, 34]. We found a high expression of *OTX2* also in PB which was expected as OTX2 is essential for the normal development of brain, cerebellum, pineal gland, and eye [3]. *OTX2* expression was even shown in adult rat pinealocytes and Trubicka et al. used it as a marker gene to distinguish PB samples from other *BCOR* expressing tumors [28, 31]. The copy number gains and high expression of *OTX2* and its downstream target *OTX3*—with the high *OTX3* expression also shown by Liu et al. before [20]—in all PB subtypes suggests an oncogenic role of OTX2 for PB tumorigenesis.

## Supplementary Information

Additional file1 (PPTX 8250 kb)

Additional file2 (XLSX 22 kb)

## Data Availability

Data will be made available upon reasonable request from the corresponding author.
