# Supplementary material for: Recurrent genetic alterations in epigenetically defined pineoblastoma subtypes
Source: Acta Neuropathol Commun. 2025 Nov 25;13:241. doi: 10.1186/s40478-025-02140-7 (PMC12648887; doi:10.1186/s40478-025-02140-7)
Supplement: Supplementary file 1 — Additional file1 (PPTX 8250 kb) [file 40478_2025_2140_MOESM1_ESM.pptx]

## Slide 1
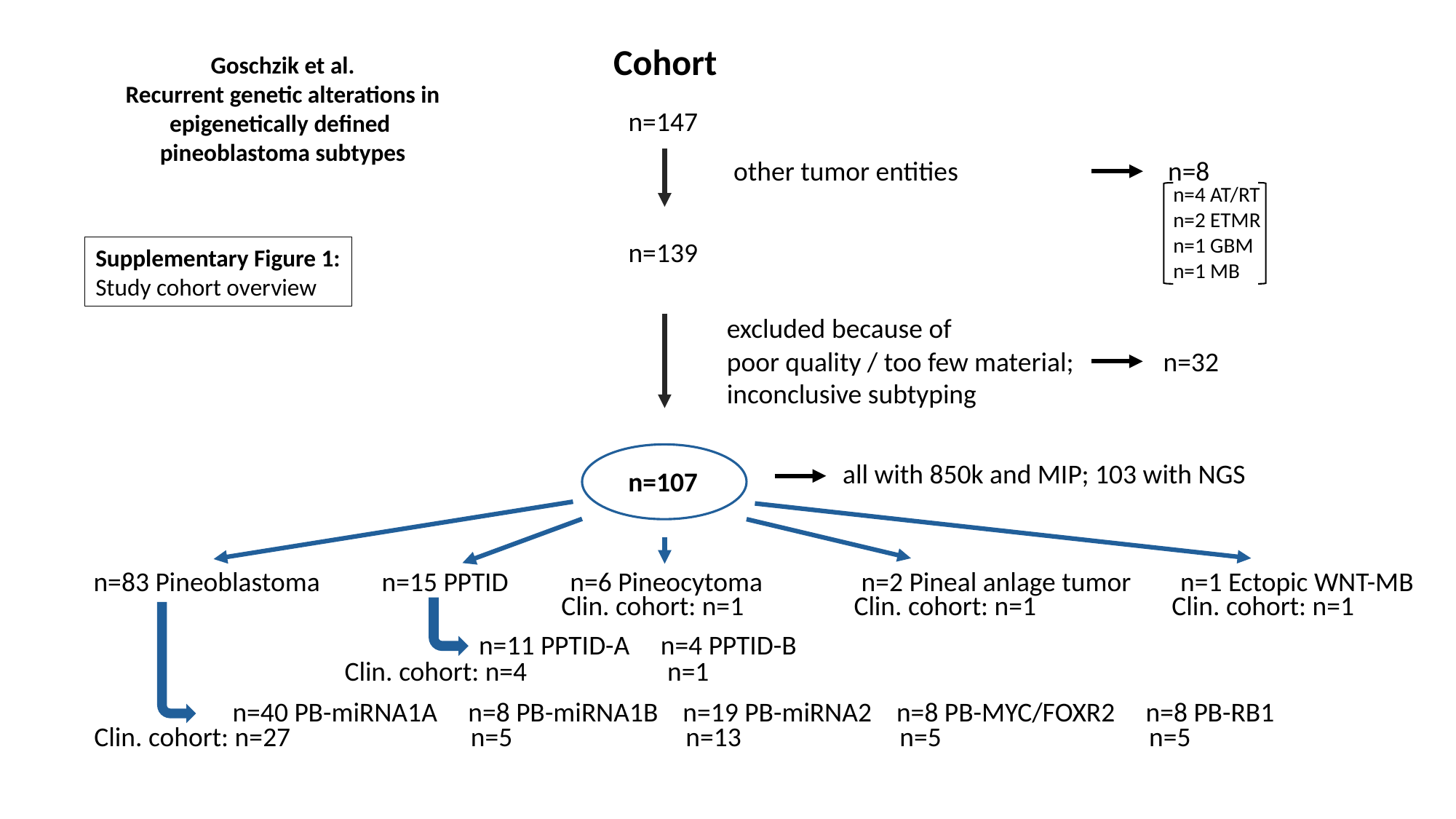

Cohort
Goschzik et al.
Recurrent genetic alterations in epigenetically defined
pineoblastoma subtypes
n=147
n=139
n=107
other tumor entities n=8
n=4 AT/RT
n=2 ETMR
n=1 GBM
n=1 MB
Supplementary Figure 1:
Study cohort overview
excluded because of
poor quality / too few material; n=32
inconclusive subtyping
 all with 850k and MIP; 103 with NGS
n=83 Pineoblastoma n=15 PPTID n=6 Pineocytoma n=2 Pineal anlage tumor n=1 Ectopic WNT-MB
Clin. cohort: n=1PPT Clin. cohort: n=1PP Clin. cohort: n=1PPT T
 n=11 PPTID-A n=4 PPTID-B
Clin. cohort: n=4 PPTID-A n=1
n=40 PB-miRNA1A n=8 PB-miRNA1B n=19 PB-miRNA2 n=8 PB-MYC/FOXR2 n=8 PB-RB1
Clin. cohort: n=27 PB-miRNA1A n=5 PB-miRNA1B n=13 PB-miRNA2 n=5 PB-MYC/FOXR2 n=5 PB-RB1

## Slide 2
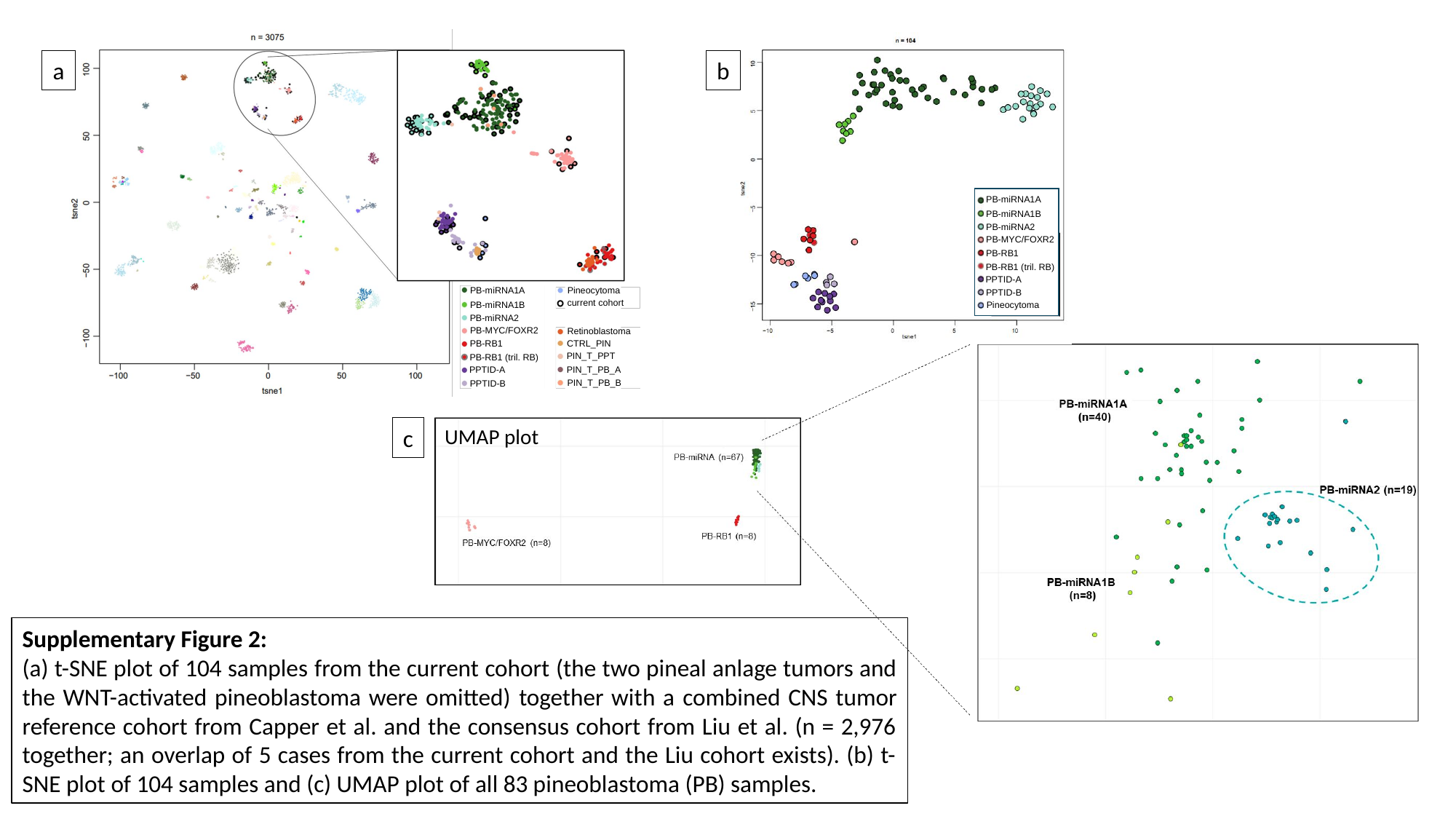

a
b
PB-miRNA1A
PB-miRNA1B
PB-miRNA2
PB-MYC/FOXR2
PB-RB1
PB-RB1 (tril. RB)
PPTID-A
PPTID-B
Pineocytoma
Pineocytoma
PB-miRNA1A
current cohort
PB-miRNA1B
PB-miRNA2
PB-MYC/FOXR2
Retinoblastoma
CTRL_PIN
PB-RB1
PIN_T_PPT
PB-RB1 (tril. RB)
PIN_T_PB_A
PPTID-A
PIN_T_PB_B
PPTID-B
c
UMAP plot
Supplementary Figure 2:
(a) t-SNE plot of 104 samples from the current cohort (the two pineal anlage tumors and the WNT-activated pineoblastoma were omitted) together with a combined CNS tumor reference cohort from Capper et al. and the consensus cohort from Liu et al. (n = 2,976 together; an overlap of 5 cases from the current cohort and the Liu cohort exists). (b) t-SNE plot of 104 samples and (c) UMAP plot of all 83 pineoblastoma (PB) samples.

## Slide 3
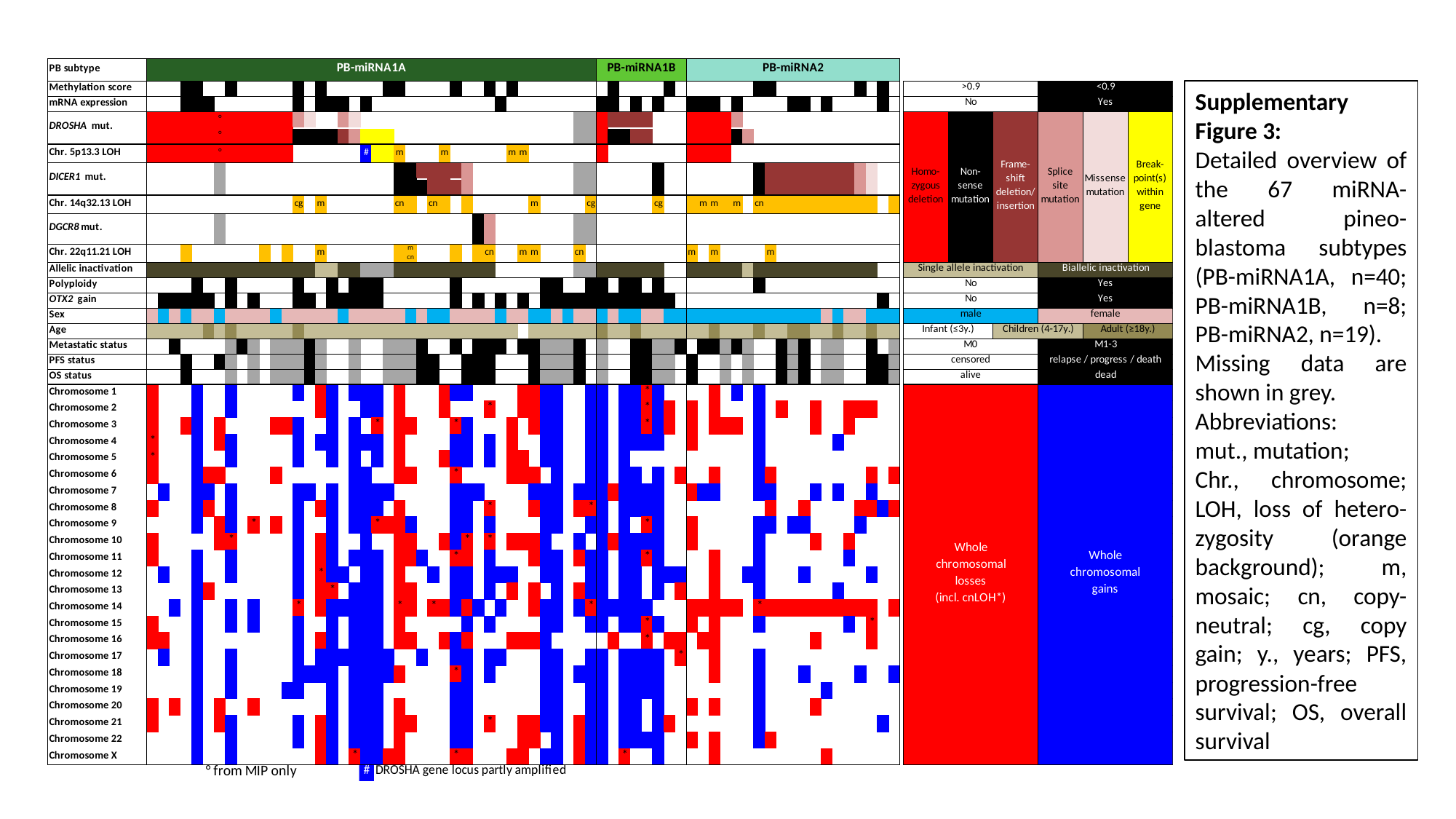

Supplementary Figure 3:
Detailed overview of the 67 miRNA-altered pineo-blastoma subtypes (PB-miRNA1A, n=40; PB-miRNA1B, n=8; PB-miRNA2, n=19).
Missing data are shown in grey.
Abbreviations:
mut., mutation;
Chr., chromosome; LOH, loss of hetero-zygosity (orange background); m, mosaic; cn, copy-neutral; cg, copy gain; y., years; PFS, progression-free survival; OS, overall survival

## Slide 4
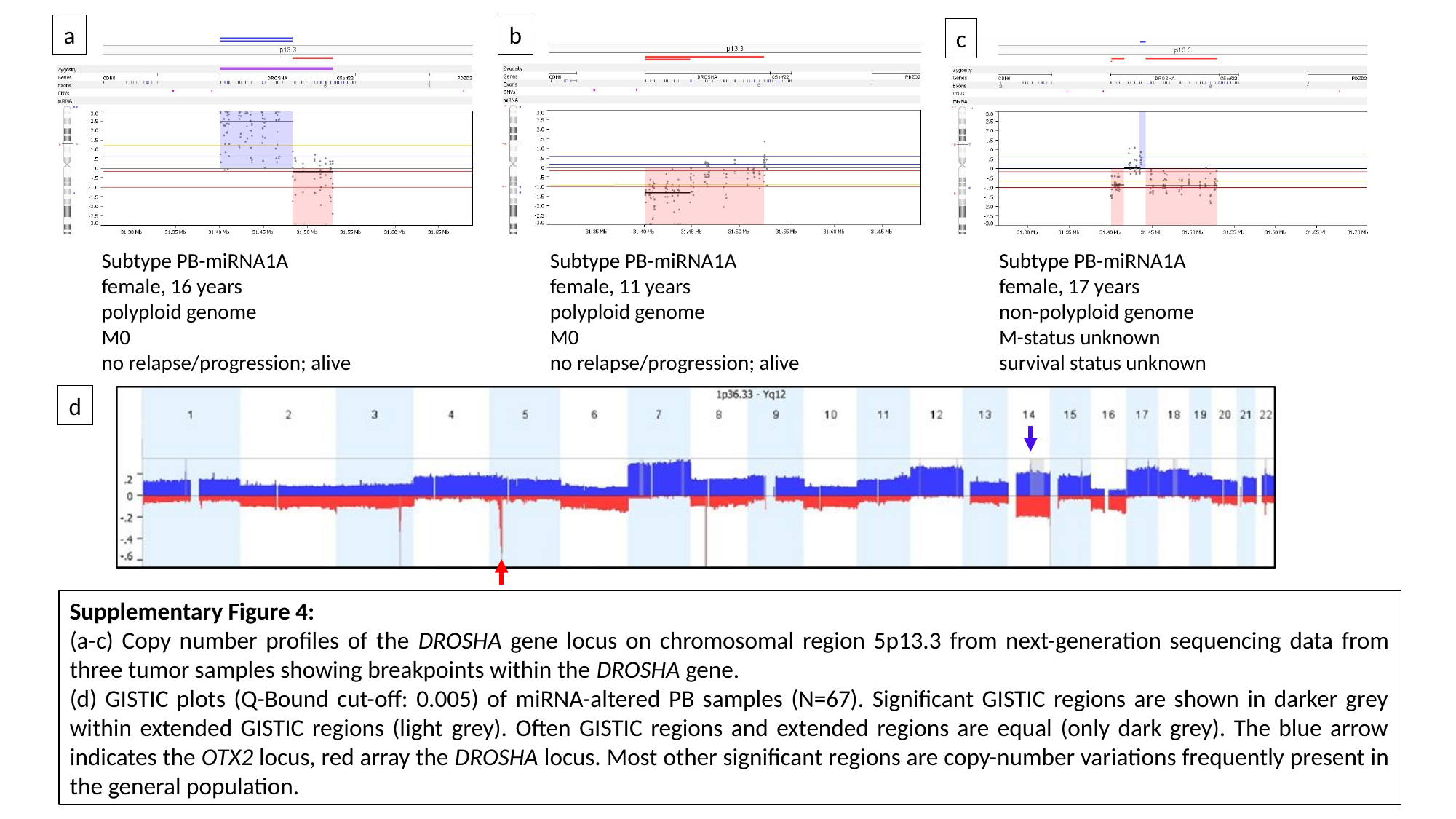

a
b
c
Subtype PB-miRNA1A
female, 17 years
non-polyploid genome
M-status unknown
survival status unknown
Subtype PB-miRNA1A
female, 16 years
polyploid genome
M0
no relapse/progression; alive
Subtype PB-miRNA1A
female, 11 years
polyploid genome
M0
no relapse/progression; alive
d
Supplementary Figure 4:
(a-c) Copy number profiles of the DROSHA gene locus on chromosomal region 5p13.3 from next-generation sequencing data from three tumor samples showing breakpoints within the DROSHA gene.
(d) GISTIC plots (Q-Bound cut-off: 0.005) of miRNA-altered PB samples (N=67). Significant GISTIC regions are shown in darker grey within extended GISTIC regions (light grey). Often GISTIC regions and extended regions are equal (only dark grey). The blue arrow indicates the OTX2 locus, red array the DROSHA locus. Most other significant regions are copy-number variations frequently present in the general population.

## Slide 5
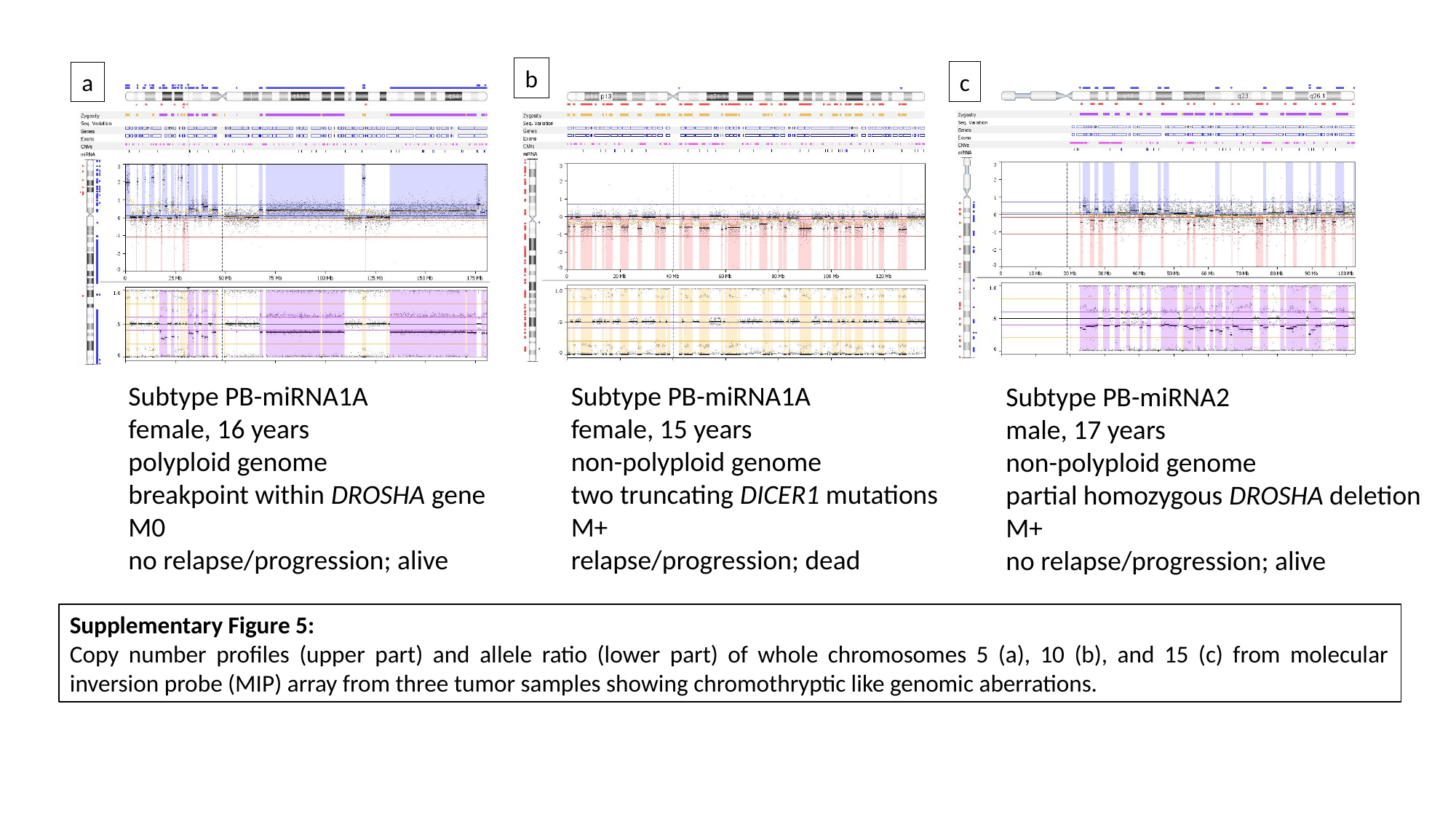

b
c
a
Subtype PB-miRNA1A
female, 16 years
polyploid genome
breakpoint within DROSHA gene
M0
no relapse/progression; alive
Subtype PB-miRNA1A
female, 15 years
non-polyploid genome
two truncating DICER1 mutations
M+
relapse/progression; dead
Subtype PB-miRNA2
male, 17 years
non-polyploid genome
partial homozygous DROSHA deletion
M+
no relapse/progression; alive
Supplementary Figure 5:
Copy number profiles (upper part) and allele ratio (lower part) of whole chromosomes 5 (a), 10 (b), and 15 (c) from molecular inversion probe (MIP) array from three tumor samples showing chromothryptic like genomic aberrations.

## Slide 6
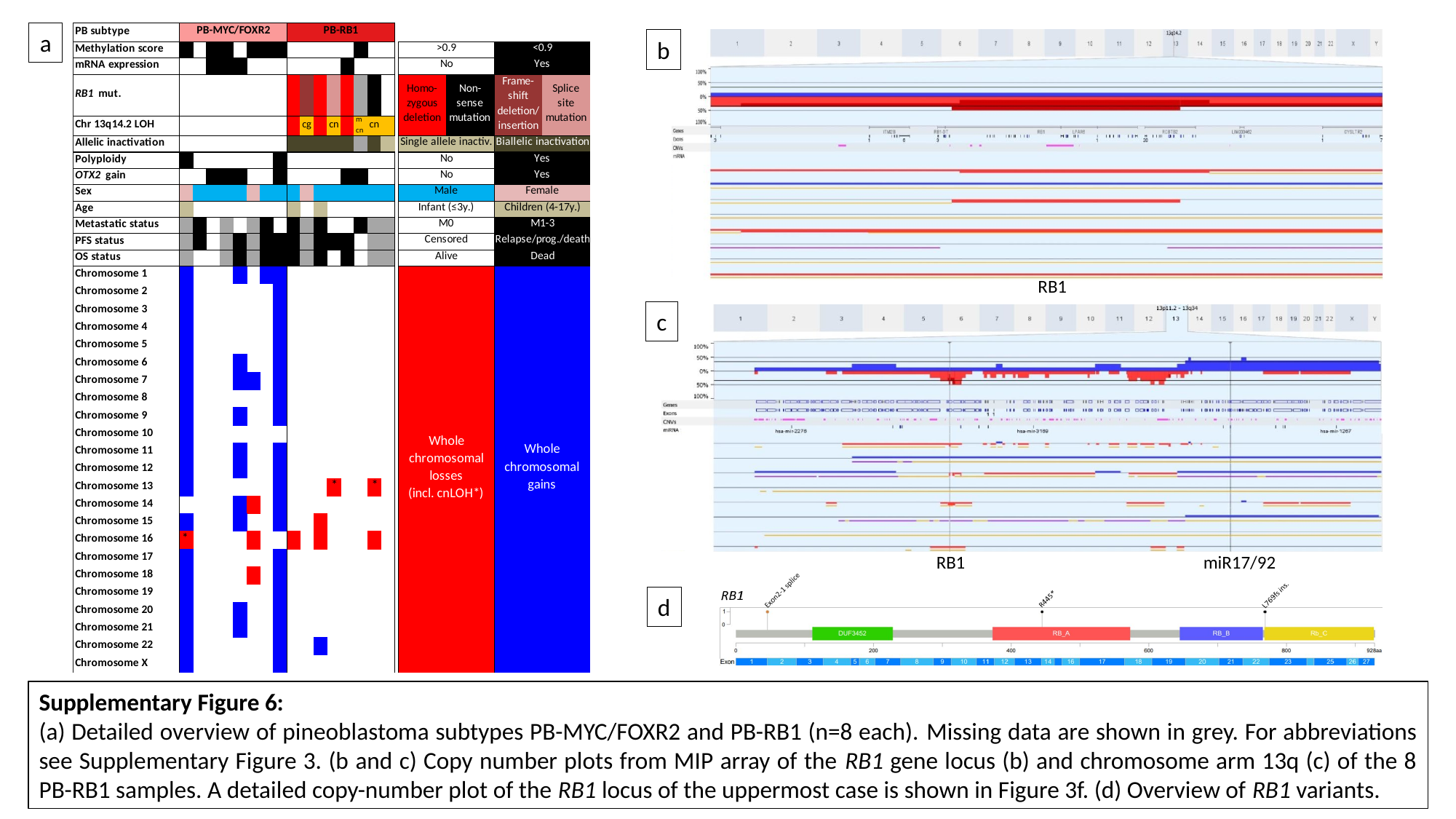

a
b
RB1
c
miR17/92
RB1
d
Supplementary Figure 6:
(a) Detailed overview of pineoblastoma subtypes PB-MYC/FOXR2 and PB-RB1 (n=8 each). Missing data are shown in grey. For abbreviations see Supplementary Figure 3. (b and c) Copy number plots from MIP array of the RB1 gene locus (b) and chromosome arm 13q (c) of the 8 PB-RB1 samples. A detailed copy-number plot of the RB1 locus of the uppermost case is shown in Figure 3f. (d) Overview of RB1 variants.

## Slide 7
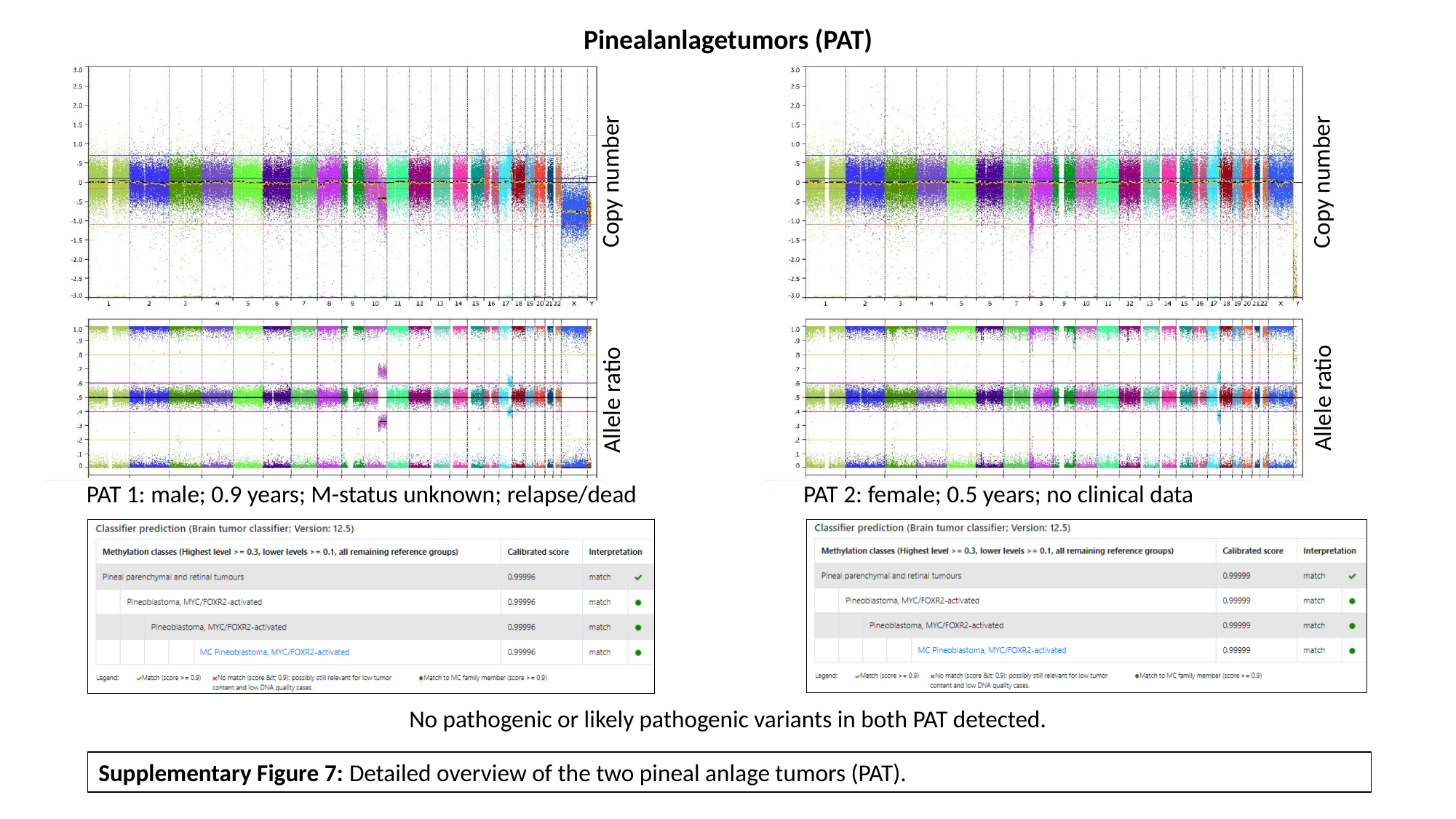

Pinealanlagetumors (PAT)
Copy number
Copy number
Allele ratio
Allele ratio
PAT 2: female; 0.5 years; no clinical data
 PAT 1: male; 0.9 years; M-status unknown; relapse/dead
No pathogenic or likely pathogenic variants in both PAT detected.
Supplementary Figure 7: Detailed overview of the two pineal anlage tumors (PAT).

## Slide 8
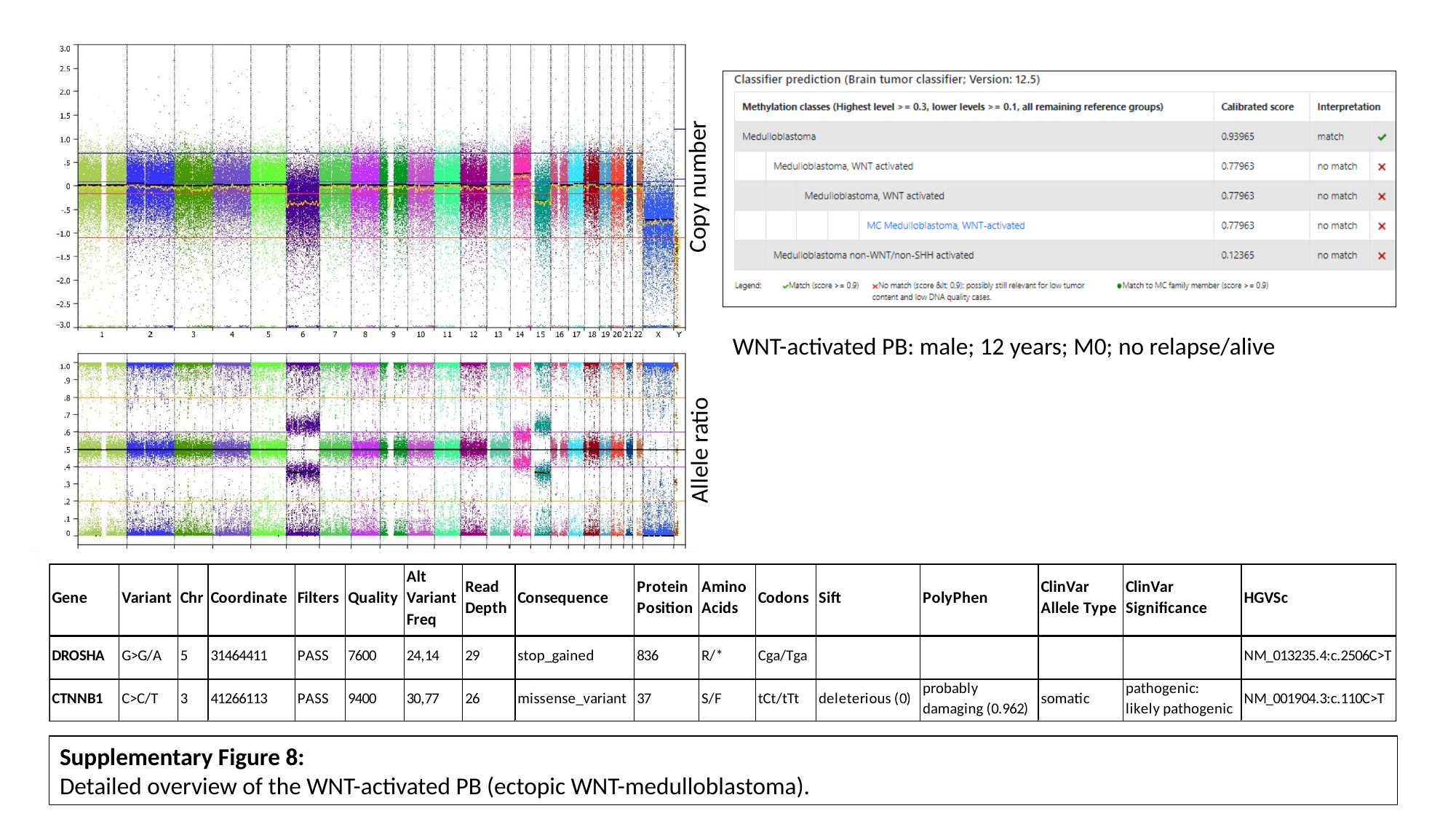

Copy number
Allele ratio
WNT-activated PB: male; 12 years; M0; no relapse/alive
Supplementary Figure 8:
Detailed overview of the WNT-activated PB (ectopic WNT-medulloblastoma).

## Slide 9
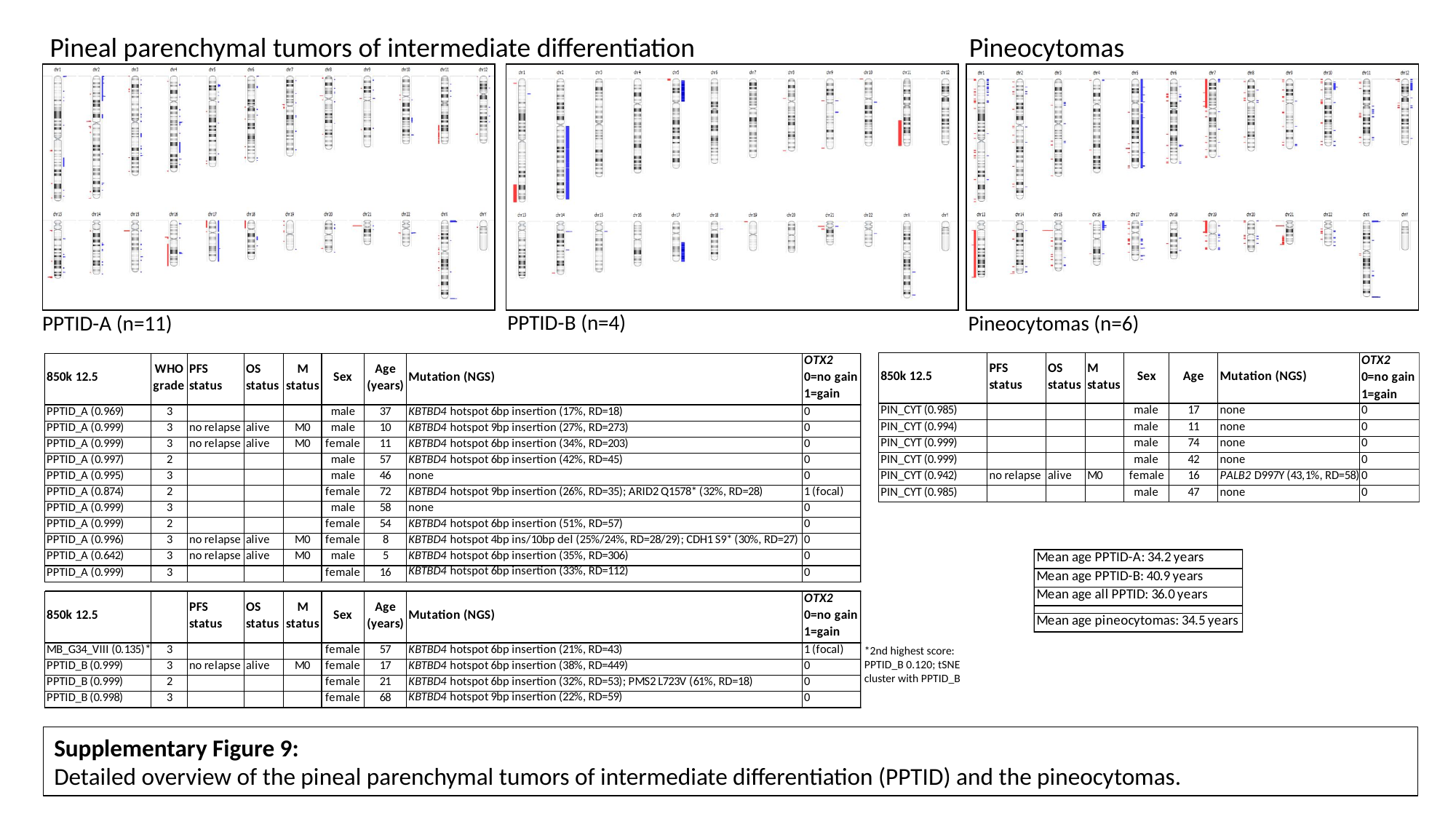

Pineocytomas
Pineal parenchymal tumors of intermediate differentiation
PPTID-B (n=4)
PPTID-A (n=11)
Pineocytomas (n=6)
*2nd highest score: PPTID_B 0.120; tSNE cluster with PPTID_B
Supplementary Figure 9:
Detailed overview of the pineal parenchymal tumors of intermediate differentiation (PPTID) and the pineocytomas.
